# Genetic Architecture of Powdery Mildew Resistance Revealed by a Genome-Wide Association Study of a Worldwide Collection of Flax (*Linum usitatissimum L*.)

**DOI:** 10.3389/fpls.2022.871633

**Published:** 2022-06-24

**Authors:** Adrien Speck, Jean-Paul Trouvé, Jérôme Enjalbert, Valérie Geffroy, Johann Joets, Laurence Moreau

**Affiliations:** ^1^Terre de Lin, Saint-Pierre-le-Viger, France; ^2^Université Paris-Saclay, INRAE, CNRS, AgroParisTech, Génétique Quantitative et Evolution - Le Moulon, Gif-sur-Yvette, France; ^3^Université Paris-Saclay, CNRS, INRAE, Université Evry, Institute of Plant Sciences Paris-Saclay (IPS2), Gif-sur-Yvette, France; ^4^Université de Paris, Institute of Plant Sciences Paris-Saclay (IPS2), Gif-sur-Yvette, France

**Keywords:** flax (*Linum usitatissimum L*.), GBS, genetic diversity, powdery mildew, genome wide association studies (GWAS)

## Abstract

Powdery mildew is one of the most important diseases of flax and is particularly prejudicial to its yield and oil or fiber quality. This disease, caused by the obligate biotrophic ascomycete *Oïdium lini*, is progressing in France. Genetic resistance of varieties is critical for the control of this disease, but very few resistance genes have been identified so far. It is therefore necessary to identify new resistance genes to powdery mildew suitable to the local context of pathogenicity. For this purpose, we studied a worldwide diversity panel composed of 311 flax genotypes both phenotyped for resistance to powdery mildew resistance over 2 years of field trials in France and resequenced. Sequence reads were mapped on the CDC Bethune reference genome revealing 1,693,910 high-quality SNPs, further used for both population structure analysis and genome-wide association studies (GWASs). A number of four major genetic groups were identified, separating oil flax accessions from America or Europe and those from Asia or Middle-East and fiber flax accessions originating from Eastern Europe and those from Western Europe. A number of eight QTLs were detected at the false discovery rate threshold of 5%, located on chromosomes 1, 2, 4, 13, and 14. Taking advantage of the moderate linkage disequilibrium present in the flax panel, and using the available genome annotation, we identified potential candidate genes. Our study shows the existence of new resistance alleles against powdery mildew in our diversity panel, of high interest for flax breeding program.

## Introduction

Flax (*Linum usitatissimum L*.) is an important industrial crop providing both stem fibers and linseeds that are used to produce textiles, edible oil, animal feed, and other industrial products such as sound and thermal insulation panels or composite materials (Jhala and Hall, 2010; Goudenhooft et al., 2019). Archaeological evidence of flax seeds and pieces of woven linen were discovered in many countries of the Fertile Crescent such as in Syria, Iran, Israel, and Cisjordania (Zohari, 2013). It has been suggested that flax was domesticated from pre-ceramic Neolithic times about 10,000 years ago first for its seeds and 1,000 years after for its fibers (Diederichsen and Hammer, 1995; Allaby et al., 2005; Fu and Allaby, 2010; Xie et al., 2018). Early domestication history of flax might also involve multiple events of domestication for capsular indehiscence, and to minimize vernalization requirements (Fu, 2011, 2012). Selective sweep (drastic loss of diversity in genomic regions under selection) analyses indicated human-involved selection for small seeds during the oil to fiber flax transition (Guo et al., 2020; Jiang et al., 2021) and also for flowering time, dehiscence, and plant architecture traits (Zhang et al., 2020).

A previous genetic study, based on 407 flax accessions genotyped by 259 SSRs, identified three major genetic groups: an oil (O) group subdivided into three sub-groups representing accessions from South Asia, Western Europe, and South America; a fiber (F) group distributed into two sub-groups, namely, North America and Eastern Europe; and an admixured (OxF) group including accessions from North America and Europe (Soto-Cerda et al., 2013). However, only a weak population structure (*F*_ST_ = 0.094) between fiber and oil flax groups was observed (with a low representativeness of fiber flax in the panel studied: 22%). Genomic analysis between fiber and oil flax identified candidate genes involved in cell-wall biogenesis or modification, auxin regulation, and fatty acid biosynthesis. Moreover, the level of differentiation between sub-groups ranged from 0.02 (*p* < 0.001, North America vs. Eastern Europe) to 0.16 (*p* < 0.001, Eastern Europe vs. South Asia). A more recent Chinese study, based on resequencing of 200 cultivated flax accessions, proposed a similar classification into three major groups according to the flax type (O, F, and OxF) and identified a closer genetic relationship between fiber (F) flax and admixture (OxF) flax groups (Guo et al., 2020). In addition, in 2020, a collection composed by 350 worldwide flax accessions and genotyped with 6,200 single-nucleotide polymorphism (SNP) markers revealed a clustering into seven sub-populations (Hoque et al., 2020). A total of six out of these seven sub-populations consisted of oil-type genotypes. The remaining sub-population consisted of mostly fiber-type genotypes. The pairwise *F*_ST_ comparison revealed a great degree of divergence between the oil sub-population originated from North America and Asia (*F*_ST_ = 0.54), as well as between the oil sub-population originated from North America and the fiber group originated from Europe (*F*_ST_ = 0.54).

Flax is affected by several diseases, but powdery mildew is one of the most prevalent in the word (Rashid et al., 1999; Stafecka et al., 2017) and in particular in France. This disease is caused by the obligate biotrophic *ascomycete Oidium lini Skoric* (imperfect stage) or *Erysiphe polygoni* (perfect stage) (Muskett and Colhoun, 1947; Aly et al., 2017). Powdery mildew is an air-borne disease that causes fiber and seed yield losses due to the presence of a layer of white mycelium on leave and stem surfaces resulting in a decreased photosynthetic activity and an acceleration of plant maturation. The expansion of winter flax farming of +180% in the last 3 years (TERRE DE LIN, pers. Com.) could be partially responsible for the increase of this disease. Flax is traditionally sown in spring, whereas winter flax is sown during the fall and harvested 1 month before the spring flax in early summer. Use of winter flax extends the period when flax is present in the fields which may favor pathogen development. Powdery mildew can be controlled using fungicides to ensure a preventive control (cyflufenamid active ingredient) or a curative control (prothioconazole-active ingredient). However, restriction of chemical spreading could occur soon in Europe, thereby accelerating the need to develop new varieties with resistance genes to effectively manage the disease.

Previous studies reported the presence of several major genes or quantitative trait loci (QTLs) for resistance to powdery mildew. A number of one dominant gene (designated Ol1) was identified in Indian oil cultivars by studying the segregation of resistance in F2 populations (Singh and Saharan, 1979; Basandrai and Basandrai, 2000). Later, one dominant gene (designated Pm1) was identified from Canadian oil cultivars, and two additional dominant genes were found in European oil cultivars (Atalante and Linda) using similar genetic approaches (Rashid and Duguid, 2005). In 2013, several powdery mildew resistance QTLs have been reported on chromosomes 1, 7, and 9 using a F2 population of 300 individuals generated from a cross between the susceptible oil cultivar Norman and the resistant oil cultivar Linda. These QTLs explained 97% of the phenotypic variation (Asgarinia et al., 2013).

Bi-parental QTL linkage mapping is a powerful method to identify genetic regions related to the trait of interest within the studied population (Broman, 2001). However, it requires the development of a large segregating population for the trait of interest, and results cannot be extrapolated to other populations, thus limiting its use. Genome-wide association study (GWAS) allows mapping QTLs underlying a trait of interest in germplasm of diverse origin. The basic principle in GWAS is to test the association between each marker and a phenotype of interest that has been scored across unrelated individuals of a diversity panel (Mitchell-Olds, 2010; Alqudah et al., 2020). Linkage disequilibrium (LD) is the main factor that influences marker density requirement and mapping resolution in GWAS analysis. An increase of LD is observed with new mutation, mating system (self-pollination), genetic isolation, population structure, relatedness (kinship), small founder population size or genetic drift, genomic rearrangements, and selection (result of co-selection of loci during breeding for multiple traits) (Gupta et al., 2005).

The objective of this study was to better understand the genetic architecture of the resistance to powdery mildew and to identify resistance alleles that could be introgressed into elite material. In this project, we studied 311 flax genotypes distributed over the different flax types and the various climatic regions of the world. We performed a whole-genome resequencing for each accession present in the diversity panel. The reads obtained from sequencing were mapped on the latest assembly of the oil CDC Bethune reference genome (You et al., 2018) leading to 5,464,275 SNPs. We first studied population structure and evaluated linkage disequilibrium extends. This allowed us to identify new phylogenetic groups, linked with the recent breeding history of flax. The panel was phenotyped during 2 years for powdery mildew resistance. We mapped loci associated with resistance using a GWAS approach and identified candidate genes related to powdery mildew resistance.

## Materials and Methods

### Plant Materials

A panel of 311 worldwide diverse flax accessions consisting of 173 fiber flax (F), 110 oil flax (O), 7 mixed oil-fiber flax (OxF), 2 wild flax (including one *L. bienne*), and 19 unknown type flax were used in this study. Among fiber flax accessions, 7 originated from the current TERRE DE LIN breeding program. Sampled over 38 countries, the different accessions represent all continents and diverse worldwide climatic regions. Seeds of the evaluated panel were provided by the Plant Gene Resources of Canada (PGRC), the N.I.Vavilov Institute of Plant Genetic Resources (VIR), and the personal collection from TERRE DE LIN (Supplementary Figure S1 and Supplementary Table S1). To prevent potential contaminations in collection seed lots, and to check and improve genotype fixation (homogeneity), each genotype seed lot was reconstituted by single seed descent method during the year 2017 in a greenhouse.

### DNA Extraction, Library Construction, and Sequencing

Genomic DNA was extracted from 80 mg of fresh leaves using a modified DNeasy Plant Mini Kit (Qiagen), adapted to produce high-quality and quantity DNA for PCR-free sequencing. The main modified steps were the addition of sodium bisulfite and RNAse A in the lysis buffer and an additional ethanol wash step before the DNA elution step. Integrity of genomic DNA was determined by running 2 μl of each sample in 1% agarose gel, 1X TAE buffer (Thermo Fisher Scientific) for 30 min at 100 V. The DNA was stained with 1X ethidium bromide solution (Sigma-Aldrich). The molecular weight of the genomic DNA was estimated by comparison with a 1-kb DNA ladder (Invitrogen). After this step, genomic DNA was checked for quality on NanoDrop ND8000 (Thermo Fisher Scientific) and quantified on Qubit (Thermo Fisher Scientific) by Picogreen dosage. Minimum conditions required for the preparation of PCR-free libraries were as follows: a 260/230 nm ratio over 1.8, a 260/280 nm ratio over 1.8, and at least 3 μg of genomic DNA. Illumina TruSeq PCR-free libraries were carried out using the DNA Shearing M220 (Covaris) and were checked by the Fragment Analyzer (Agilent). Sequencing was performed as 150-bp paired-end reads on NovaSeq6000 (Illumina). Both library preparation and sequencing were done by GeT-PlaGe platform, Genotoul, France.

### SNP Discovery

The quality of the sequenced reads was evaluated using FastQC v0.11.2 developed by the Babraham Institute (www.bioinformatics.babraham.ac.uk/projects/fastqc/). Then, short reads were aligned with Stampy v1.0.20 (Lunter and Goodson, 2011) onto the oil flax reference genome CDC Bethune (You et al., 2018). Stampy can map reads from the highly divergent individuals present in our diversity panel to a reference genome by allowing sequence variations such as insertions or deletions. The alignments were coordinate sorted and converted to the Binary Alignment Map (BAM) format and then filtered for their mapping quality (MAPQ Phred-score > 80) using SAMtools v1.10 (Ramirez-Gonzalez et al., 2012). Finally, the SNP calling was performed using VarScan v2.3.9 (Koboldt et al., 2012) in Variant Calling Format (VCF) with the following conditions: minimum of 3 reads overlapping the SNP, minimum average base quality of 20, minimum variant allele frequency of 0.8, and a *p*-value of 0.05. Locations where SNPs were detected from CDC Bethune reads mapped on the reference genome (false-positive SNPs) were excluded from the analysis with VCFtools v0.1.17 (Danecek et al., 2011).

Validation of the SNP discovery pipeline was carried out by comparing the SNP alleles detected by the pipeline with those already known in genotyping projects (TERRE DE LIN private SNPs; KASP technology, LGC) on two accessions (332 SNPs on CC109 accession and 337 SNPs on CC123 accession).

Visualization of SNP density plots was carried out using the CMplot v3.6.2 R-package (https://github.com/YinLiLin/CMplot). Private SNPs belonging strictly to fiber, oil, and wild groups were tracked using BCFtools (Danecek et al., 2021) and visualized along the 15 chromosomes using the circlize v0.4.13 R-package (https://github.com/jokergoo/circlize).

High-quality SNPs were retained (wild accessions excluded) using BCFtools for subsequent analyses based on a missing rate (MR) <10% and on a minor allele frequency (MAF) ≥ 5%.

### Principal Component Analysis, Population Structure, and Linkage Disequilibrium Analysis

Prior to population structure analysis, we first imputed the missing data using Beagle v5.1 (Browning, 2008; Browning and Browning, 2016) with default parameter settings. As principal component analysis (PCA) and population structure analysis are affected by linkage disequilibrium (LD) between markers, we pruned the genotyping matrix using PLINK's –indep-pairwise function with a scanning window of 50 SNPs (step size of 5 SNPs) and a LD threshold of r^2^ > 0.4 (Purcell et al., 2007).

We performed two analysis of the genetic structure of the sampled flax panel: (i) a PCA using the “prcomp” R-function; (ii) a discriminant analysis of principal components (DAPC) using the R-package adegenet v2.1.3 (Jombart et al., 2010). For DAPC, the optimal number of selected principal components in the PCA step was based on the a-score criterion. In the K-means clustering step, the optimal number of clusters k was inferred after exploring k = 3–7, and considering the Bayesian information criterion (BIC) to assess the best supported model. To determine the most likely k-value in this flax panel, we also considered the correspondence between identified groups and breeder's knowledge of the varieties present in the diversity panel. The membership probabilities of each individual to the k different groups were obtained from DAPC analysis.

Pairwise *F*_ST_ between sub-populations identified by DAPC were computed with a R-script using the method proposed by Weir and Cockerham (1984).

Linkage disequilibrium distribution between pairwise SNPs across the whole genome was evaluated from a random sub-sample of 20,000 SNPs of the imputed matrix without the wild accessions using the estimator of Rogers and Huff (Rogers and Huff, 2009) with the Rutilstimflutre v0.170.0 R-package (https://github.com/timflutre/rutilstimflutre) according to the following equation:


$$\begin{array}{l} {r^{2}}_{RH}=\frac{{cov\left(g_{i},g_{j}\right)}^{2}}{var\left(g_{i}\right)\times var\left(g_{j}\right)} \end{array}$$

where g_i_ and g_j_ are the genotypes at SNP i and j, respectively, coded as 0 for the reference allele present in CDC Bethune and 1 for the alternative allele.

### Field Phenotyping

The flax panel, sown in May 2018 and 2019, was phenotyped for key agronomic traits under natural Oï*dium lini* infection in the field during the months of July 2018 and 2019 at La Gaillarde, France (49°50'11.8“N 0°51'33.6”E, 43-m altitude, 800-mm annual rainfall).

The experimental setup was sown in three replicates according to a randomized complete block design (RCBD). In 2018, each block consisted of 10 columns and 33 rows with one susceptible (Drakkar, fiber type, TERRE DE LIN) and one tolerant genotype (Bolchoï, fiber type, TERRE DE LIN) per column as controls for disease spread in the field (sowing in 2019 was done in 5 columns and 65 rows). Trial plots were composed of a 2-m-long double-row with around 100 plants per row, with a row spacing of 20 cm.

Disease severity (DS) was scored as the percentage of infected leaf area (i.e., leaf area presenting sporulation) following a scale from 1 to 9 (1 = 0–5%; 9 = 75–100%). DS was recorded three times in 7-day intervals over the three replicates during the flowering period. DS was used to calculate the area under the disease progress curve (AUDPC) using the following formula proposed by Shaner and Finney (1977):


$$AUDPC=\sum_{i=1}^{n}\left(\frac{DS_{i+1}+DS_{i}}{2}\right)\times (t_{i+1}-\;t_{i})$$

where DS_i_ = powdery mildew severity on the i^th^ date, t_i_ = the i^th^ day and n = number of times on which DS was recorded.

### Statistical Analysis of the Phenotypic Data

The powdery mildew phenotyping data collected in 2018 and 2019 were analyzed after first checking for residual normality and error variance homogeneity at each date used for the calculation of AUDPC. We explored spatial and scoring effects on AUDPC through a mixed-model including genotype, row, and column factors modeled as random effects and the order of plot phenotyping in the field (modeling the bias in decreasing attentiveness) as a fixed effect. To correct for spatial heterogeneities, each year was analyzed separately using the SpATS v1.0–11 R-package (Rodríguez-Álvarez et al., 2018). Spatial effects were modeled on a row and column basis by specifying the P-spline ANOVA algorithm (Lee et al., 2013), with the number of segments set to the respective number of rows and columns in our RCBD experimental design. Fitted values generated by the SpATS model were used for subsequent analysis. We calculated broad-sense heritability using the “getHeritability” SpATS function, which returns the generalized heritability proposed by Oakey et al. (2006).

### Genome-Wide Association Study Analysis

Genome-wide association study analysis was done using the genotyping of 1,693,910 SNPs of 286 genotypes and the AUDPC phenotypic data corrected for spatial heterogeneities, separately for the years 2018 and 2019. Wild flax, the CDC Bethune variety used for mapping reads, and genotypes belonging to the AS-ME cluster (group of small size, poorly related to the other groups and which exhibited long distance LD, cf results on panel structure and LD extent) were excluded from GWAS. Analyses were performed by applying a mixed linear association model (MLM) with a leave-one-chromosome-out (LOCO) approach as implemented in GCTA software v1.93 (Yang et al., 2011, 2014), taking into consideration the panel structure by the kinship matrix and by including the DAPC clustering as quantitative covariates in the model. For each SNP, the MLM model was as follows:


$$\begin{array}{l} Y=\mu 1+Qc+bx+g+\;\varepsilon \end{array}$$

where Y is a vector of the phenotypes, μ is the mean, 1 is a vector of ones, Q is the matrix of covariates issued from DAPC, c if the corresponding vector of covariate fixed effects, b is the additive fixed effect of the SNP tested for association, x is a vector of the SNP genotypes coded as 0, 1 or 2, corresponding to the copy number of the reference allele, g is the vector of random polygenic effect, and ε is a vector of residuals. The residuals were assumed to be independent and normally distributed ε ~ *N*(0,I$\sigma_{\text{e}}^{2}$) with $\sigma_{\text{e}}^{2}$ being the residual variance. The random polygenetic effects were assumed to follow a normal distribution with g ~ *N*(0, A$\sigma_{\text{g}}^{2}$), where A is the genomic relationship matrix for which the chromosome of the SNP tested is ignored and $\sigma_{\text{g}}^{2}$ is the genetic variance. The genetic relationships among all of the 286 individuals were estimated in GCTA with the following equation:


$$A_{jk}=\frac{1}{N}\sum_{i}^{\;}A_{ijk}=\{\begin{array}{c} \frac{1}{N}\sum_{i}^{\;}\frac{\left(x_{ij}-2p_{i})(x_{ik}-2p_{i}\right)}{2p_{i}\left(1-p_{i}\right)},\;j\neq k\\ 1+\frac{1}{N}\sum_{i}^{\;}\frac{x_{ij}^{2}-\left(1+2p_{i}\right)x_{ij}+2p_{i}^{2}}{2p_{i}\left(1-p_{i}\right)},\;j=k \end{array}$$

where *A*_j*k*_ is the calculated genetic relationship value for individuals *j* and *k*; *N* is the number of SNPs shared between two individuals on all the chromosomes except the one of the tested SNP; *x*_i*j*_ is the number of copies of the reference allele for the i^th^ SNP of the j^th^ individual; *p*_i_ is the frequency of the reference allele for locus i.

The quantile–quantile (QQ) and Manhattan plots were visualized using the “qqman” v0.1.4 R-package (Turner, 2014). We applied the Benjamini–Hochberg false discovery rate (FDR) approach (Benjamini and Hochberg, 1995), which controls the expected proportion of false positives among all signals with a FDR value below a fixed threshold, to determine significance. Subsequently, we determined significant trait associations using the FDR threshold of q = 0.05. In addition to the FDR threshold, the Bonferroni significance threshold was also plotted on the graphs according to the following formula: “–log_10_ (α/ number of SNPs) with α = 0.05.” We defined as QTL chromosome regions where significant association signals were detected.

### Candidate Gene Identification

Candidate loci were explored using a combination of GWAS *p*-values, local linkage disequilibrium, and gene annotation using the GFF3 file of the *Linum usitatissimum* genome. This information was summarized for each candidate loci using the LocusZooms R-package (https://github.com/Geeketics/LocusZooms) and the LDBlockShow tool (https://github.com/BGI-shenzhen/LDBlockShow). An approximate flanking region of 20 to 100 kb was explored for each significant SNP and adjusted according to the extent of local linkage disequilibrium with the candidate SNP (considering regions with r^2^ ≥ 0.2 with the significant SNP).

## Results

### Over 5 Million SNPs Discovered

Sequencing of the flax panel generated approximately 2.5 Tb of high-quality paired-end reads using the Illumina NovaSeq6000 sequencer, of which 95.9% of the reads aligned with the CDC Bethune reference genome, including 69% of properly mapped reads. No significant difference was observed between fiber type and oil type both on mapped reads and on properly mapped reads: 95.8% (F) / 96.2% (O) and 68.6% (F) / 69.6% (O), respectively. The average genomic depth coverage obtained in our flax panel is about 15X.

Single-nucleotide polymorphism discovery and scoring, carried out on a panel of 311 genotypes, allowed us to obtain a genotyping matrix containing 5,464,275 SNPs. To evaluate the SNP detection pipeline, we compared a set of 337 KASP genotyping assays (competitive allele-specific PCR, LGC) used at TERRE DE LIN with those newly detected by the pipeline on one fiber flax and one oil flax accessions. We found 92% of the KASP SNP markers using the bioinformatic pipeline, and all SNPs showed the expected alternative allele (Supplementary Figure S2).

**Figure 1:**
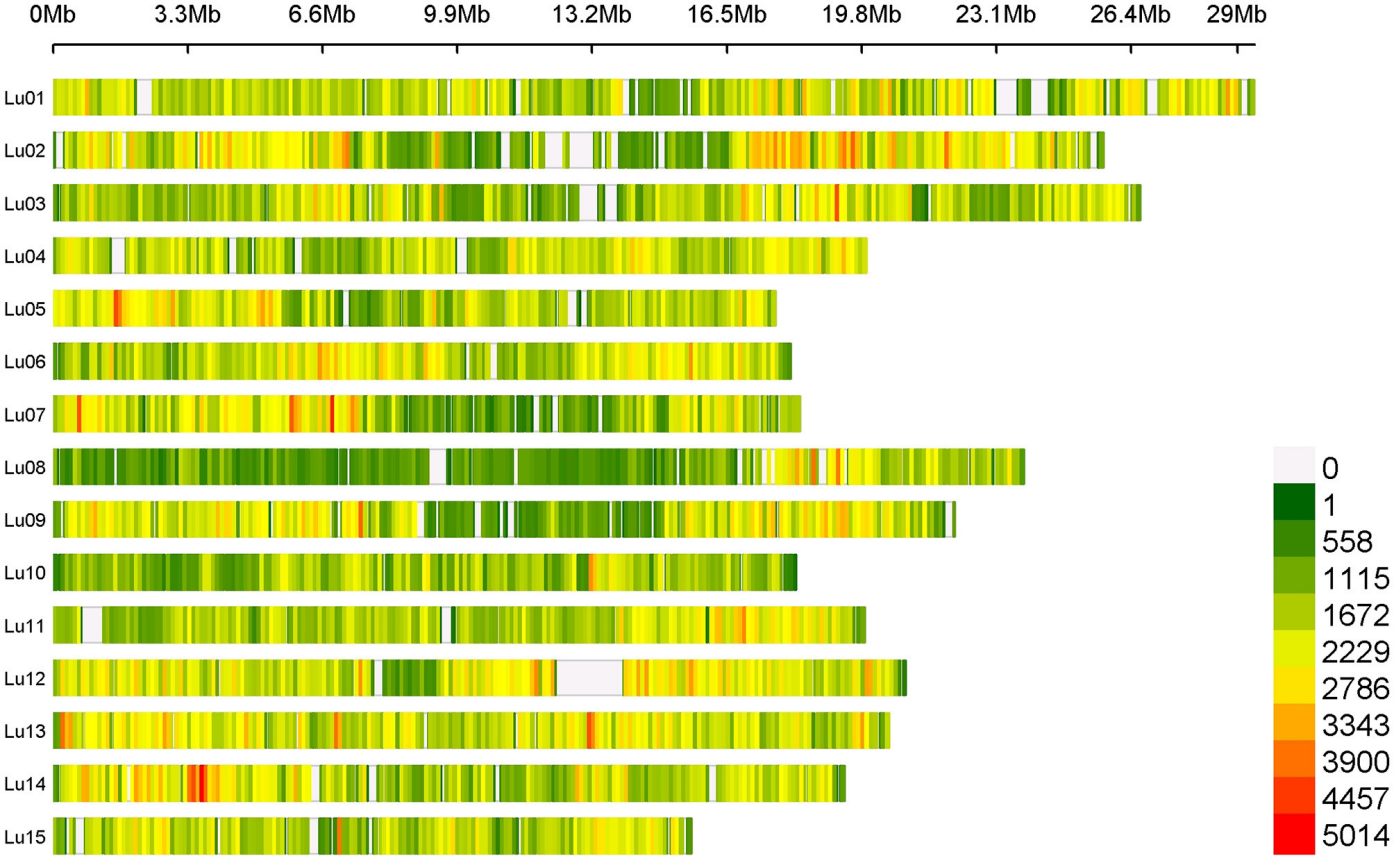
SNPs density on 15 chromosomes among the entire flax panel containing 5,464,275 SNPs per 100 kb window.

Average SNP density contained in our flax panel is about 17.28 SNPs/kb. However, this density varied depending on chromosomes and positions along chromosomes with a higher density observed on the chromosomal arms than in the central positions (centromeres). Chromosomes 8 and 13 presented the extreme SNP densities with 13.27 and 23.65 SNPs/kb, respectively (Figure 1).

**Figure 2:**
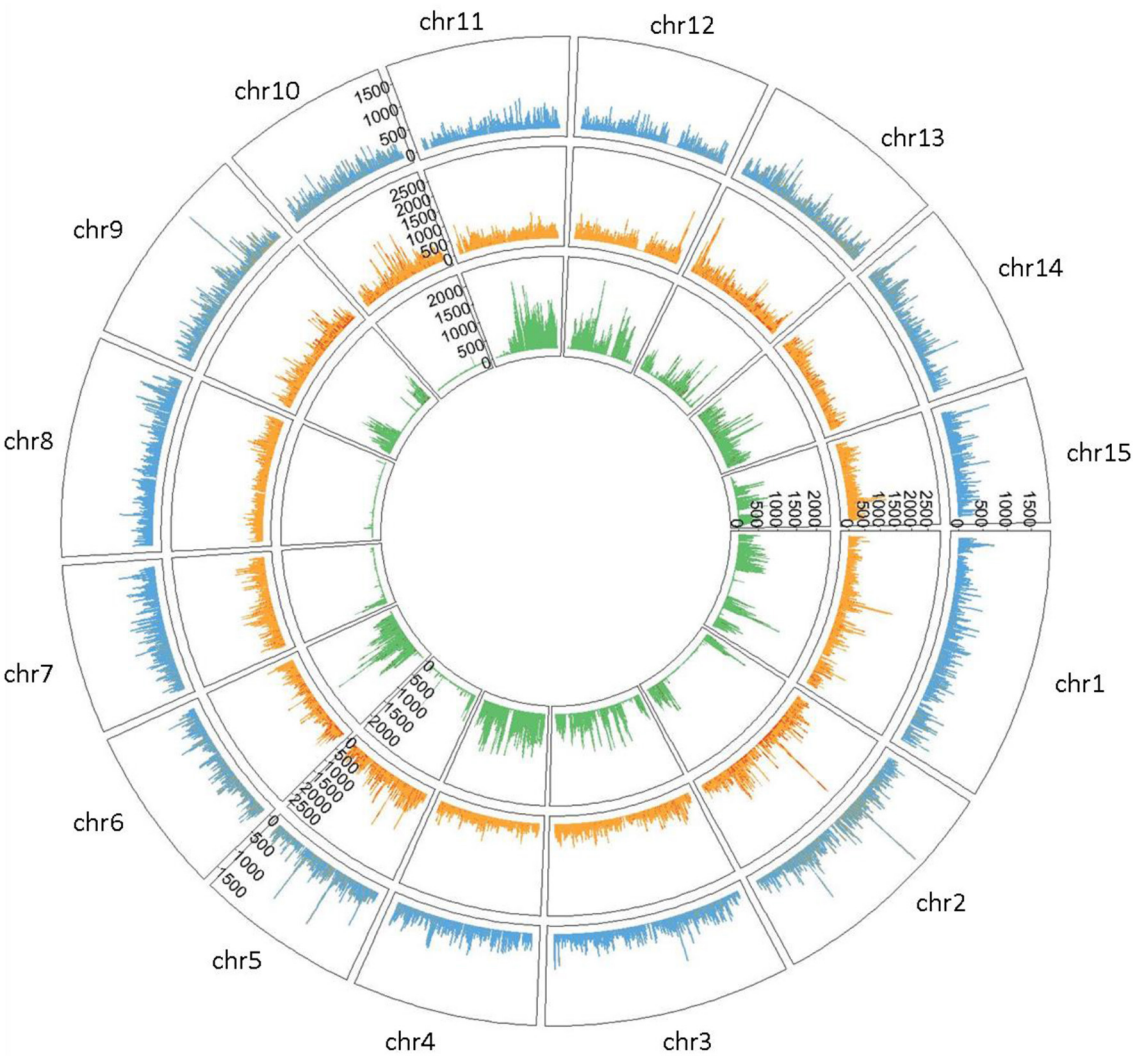
Circleplot of private SNPs by flax type (Fiber type colored in blue, Oil type in orange and Wild type in green) with a 100 kb binwidth.

Private SNPs were identified based on the flax type. We counted 722,861 private SNPs in fiber flax (13.23% of total SNPs detected), 1,058,862 in oil flax (19.38%), and 746,115 in wild flax (13.65%) (Supplementary Table S2) with a greater density on chromosomes 1 and 2 for both fiber and oil flax, and on chromosomes 4 and 11 for wild flax (Figure 2).

The occurrences of bi-allelic SNP transition were greater than those transversions by a ratio of 1.96 (Supplementary Table S3). Both A/G and C/T transitions occurred in similar frequencies (i.e., A/G 33.10 and C/T 33.16%), whereas the frequencies of the four transversions were as follows: A/T 9.20, A/C 9.02, G/T 9.01%, G/C 6.51%.

After filtering the SNPs using the MAF and missing data criteria, the genotyping matrix contained 1,693,910 high-quality SNPs (wild accessions were excluded).

### Population Structure and Clustering of Cultivated Flax

**Figure 3:**
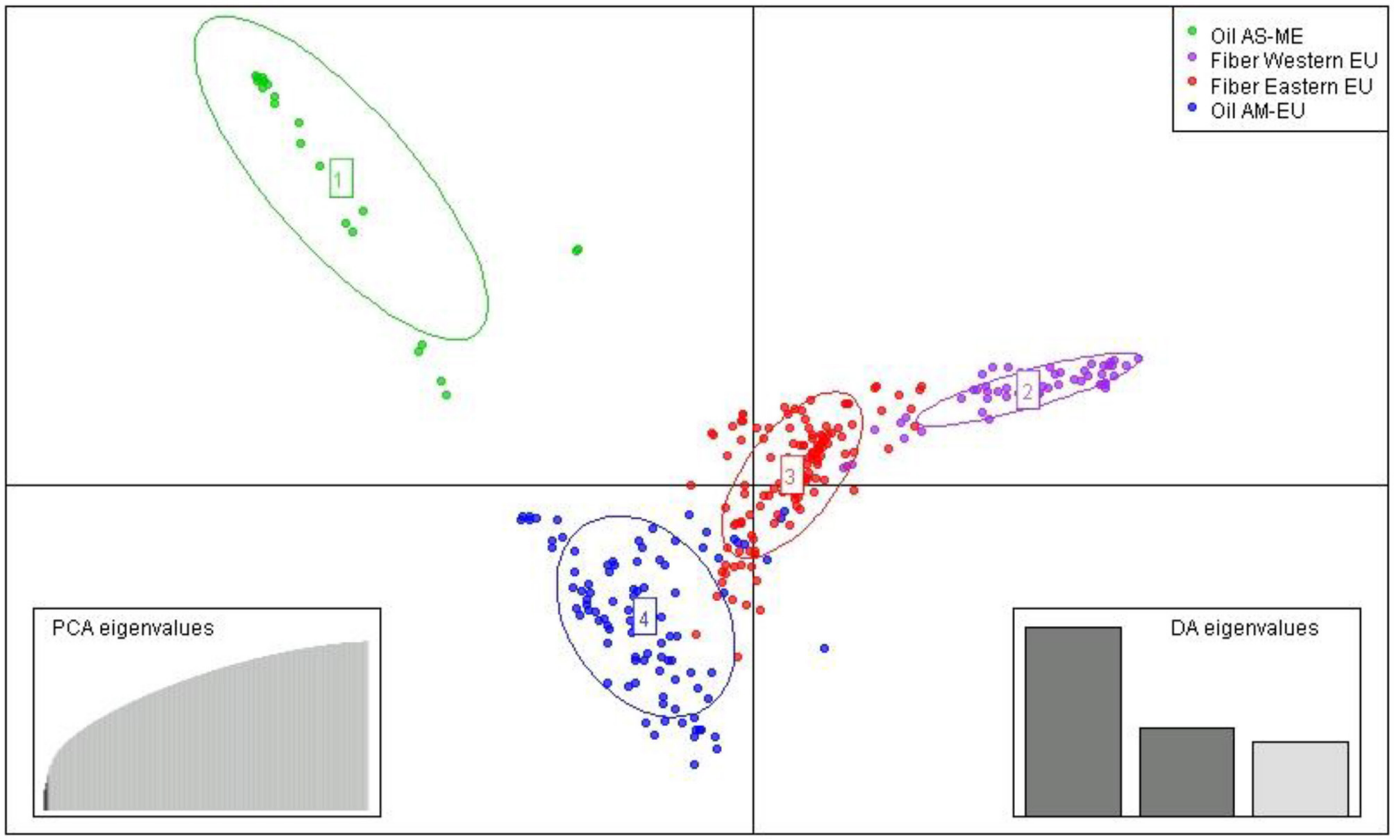
Scatter plot from DAPC (AM, America; EU, Europe; AS, Asia; ME, Middle-East).

**Figure 4:**
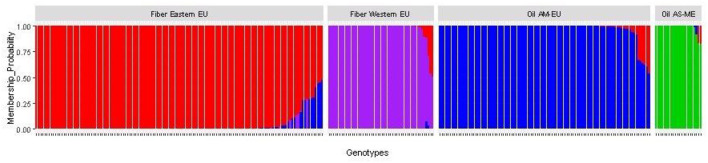
Membership probability of the flax panel for k = 4 (AM, America; EU, Europe; AS, Asia; ME, Middle-East).

After imputation of missing values, the high-quality SNP matrix containing 1,693,910 SNPs was pruned for both population structure and clustering analysis, resulting in a matrix containing 103,249 SNPs. Based on the optimal a-score plot (Supplementary Figure S3), we retained 3 PCA components that accounted for 72% of the genetic variation to produce the discriminant analysis of principal component (DAPC) scatterplot (Supplementary Figure S4). The optimal number of clusters k = 4 was inferred after exploring k = 3–7. DAPC distinguishes two fiber flax and two oil flax clusters. The first DAPC axis (vertical axis) discriminates fiber vs. oil flax, whereas the second DAPC axis (horizontal) separates the flax types according to the geographic sub-region of the world (Figure 3, refer to PCA plot in Supplementary Figure S5). Oil flax clusters are composed by 98 flax originated from America and Europe (mean membership probability = 98.6%) and 22 flax originated from Asia and Middle-East (mean membership probability = 92.7%). Fiber clusters are composed by 137 flax originated from Eastern Europe (mean membership probability = 96.2%) and 51 flax originated from Western Europe (mean membership probability = 96.1%) (Figure 4). The fiber Eastern-EU cluster shows some individuals in admixture with fiber Western-EU and oil AM-EU clusters (Supplementary Table S4).

The smallest coefficient of genetic differentiation (*F*_ST_) was observed between fiber groups (*F*_ST_ = 0.0347), whereas the highest *F*_ST_ was observed between the Asian/Middle-Eastern oil group and the two fiber groups (*F*_ST_ = 0.1598) (Supplementary Table S5).

### Genomic Distribution of Linkage Disequilibrium in Cultivated Flax

**Figure 5:**
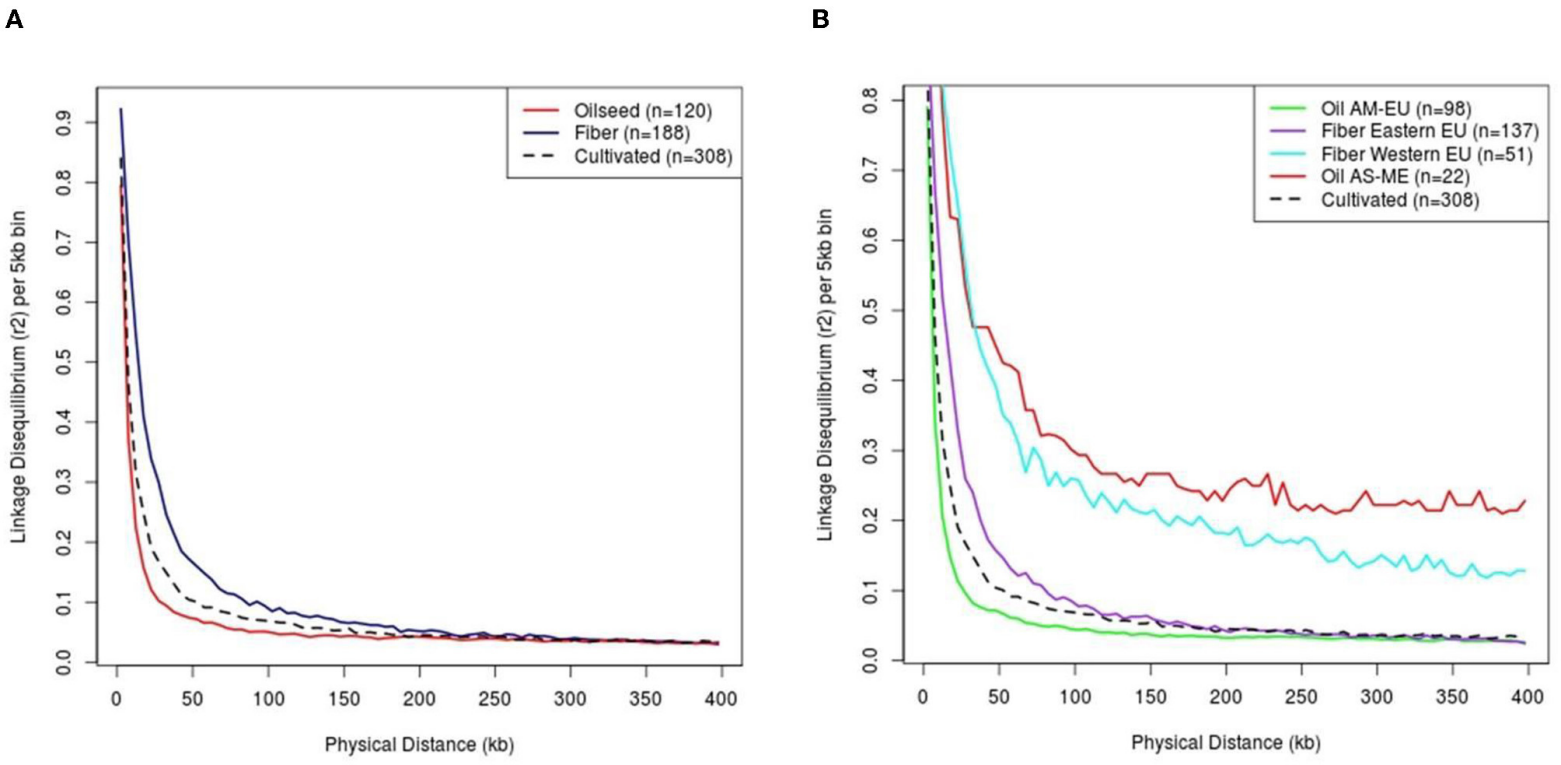
Linkage disequilibrium by flax type **(A)** and among sub-populations **(B)**. Cultivated flax corresponds to the sum of oil and fiber flax (i.e. excluding wild type). AM, America; EU, Europe; AS, Asia; ME, Middle-East.

The linkage disequilibrium decay has been studied on a random sub-sample of 20,000 SNPs and strongly varied depending on the flax type considered (Figure 5A). The extent of the observed LD in cultivated flax is around 20.43 kb for a r^2^ threshold of 0.2, with a lower LD extent in the oil cluster than in the fiber cluster (11.82 and 39.57 kb, respectively). On the same principle, the decline of LD can be measured on the subpopulations identified previously: The extent of LD is the lowest in oil AM-EU cluster (11.26 kb) and the highest in oil AS-ME cluster (threshold 0.2 not reached) (Figure 5B). Based on this result, we decided to remove from GWAS the 22 genotypes from the AS-ME group to avoid false QTL detection due to long-range LD.

### Powdery Mildew Phenotyping Under Natural Infection

Very significant disease development occurred over the 2 years, with maximum severity reaching to a score of 9 for the most susceptible lines and to a score of 1 for the most resistant lines. Statistical analysis of disease scores obtained in 2018 and 2019 showed that the local environment had a significant effect on powdery mildew resistance in the panel, in particular, the date of phenotyping (Supplementary Figure S6) and the position (row and column) of the genotype in the block. To integrate these effects along the plant cycle, we therefore decided to calculate the AUDPC based on the phenotyping dates carried out during the year and to adjust them with the R-package SpATS to correct for spatial heterogeneities in natural infection of powdery mildew in the field (Supplementary Figure S7). In 2018, the field assessment of powdery mildew resistance in the flax collection replicated in 3 blocks showed a very broad range of AUDPC from −60.52 to 45.85, even broader than the range of the two controls (−4.80–15.65 for the resistant and the susceptible reciprocally). In 2019, a similar range was observed ranging from −31.59–19.87 but with a shift from the resistant control toward a highest susceptibility genotype (4.86 for the tolerant control and 19.41 for the susceptible control). A Pearson correlation coefficient of 79% was found between 2-year data (Supplementary Figure S8 and Supplementary Table S6). The heritability (H^2^) values for AUDPC were high and equal to 0.94 for both 2018 and 2019 (Supplementary Table S7).

### Genetic Loci Associated With Resistance to *Oïdium lini via* GWAS

**Figure 6:**
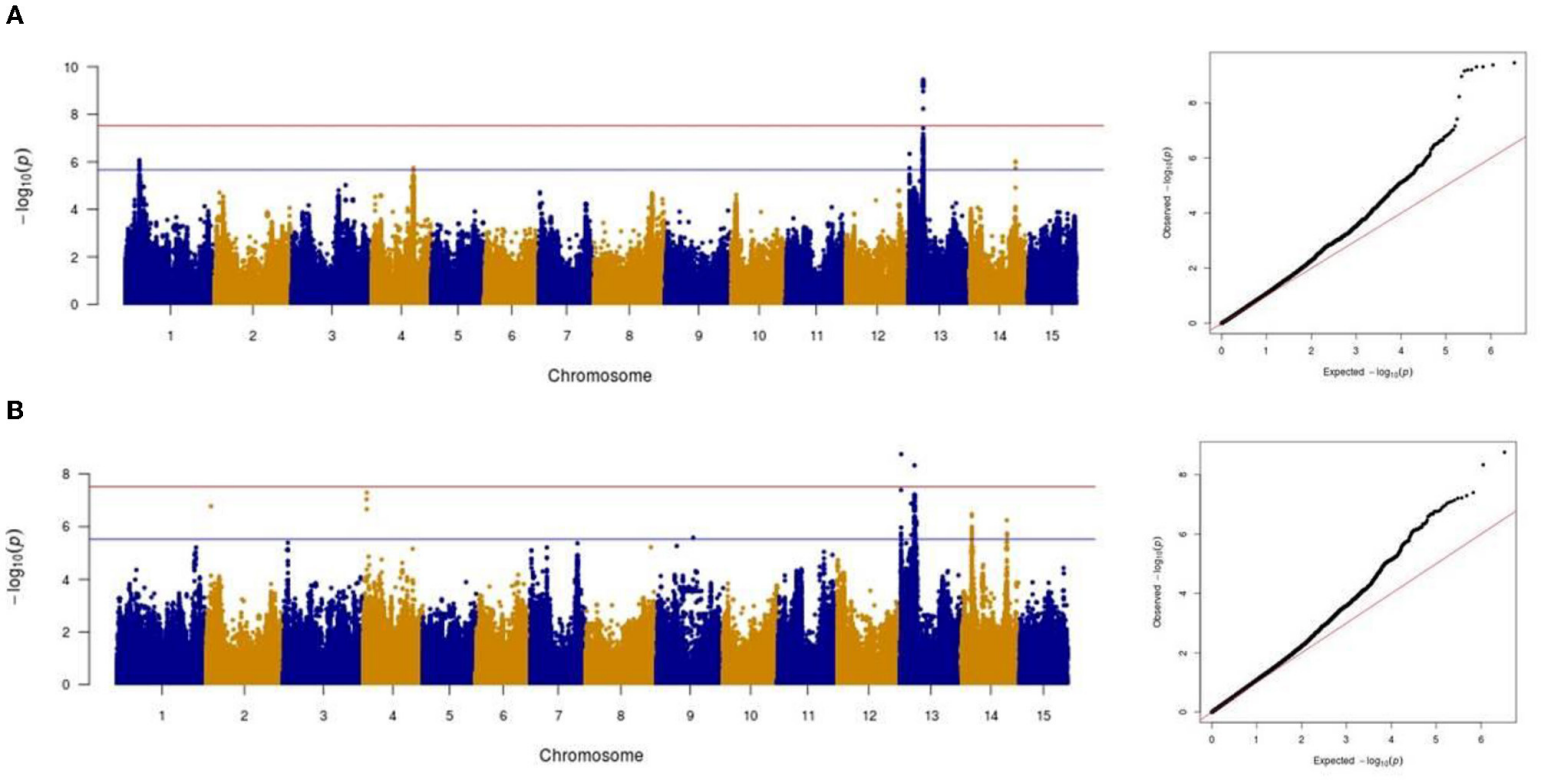
Manhattan and Quantile-quantile plots showing the significant markers detected by mixed linear model for powdery mildew in 2018 **(A)** and in 2019 **(B)**. The red horizontal line represents the Bonferroni adjusted genome-wide significance threshold (á = 0.05/1,693,910 = 2.95e-08) and the blue horizontal line the FDR threshold (*q* = 0.05).

Association analysis was conducted using 1,693,910 SNPs on a panel of 286 flax genotypes, for their AUDPC phenotypic data, to identify the genetic loci governing the resistance to *Oidium lini* under natural infection. The results obtained with the MLM model and LOCO option implemented in GCTA were visualized as Manhattan plots (Figure 6A for the year 2018 and Figure 6B for the year 2019). In total, eight significant association peaks (further referred to as QTLs) were detected at the FDR threshold of –log (p) = 4.86 in 2018 and 4.62 in 2019 and located on chromosomes 1, 2, 4, 13, and 14 (Table 1). QTL_13b and QTL_14b were significant in both 2018 and 2019. QTL_13b explained the highest proportion of the phenotypic variance (12.10%) whereas QTL_4b explained the lowest proportion (7.38%) of the phenotypic variation (in single year-based analysis). In total, detected QTLs explained 35.01 and 56.51% of the phenotypic variance in 2018 and 2019, respectively.

**Table 1:** Summary of the most significant SNPs associated with powdery mildew resistance.

**QTL name**	**Chromosome**	**SNP**	**Physical position (bp)**	**REF^**a**^**	**ALT^**b**^**	**Freq^**c**^**	**SNP effect^**d**^**	**Standard error**	**P-value**	**PVE^**e**^ (%)**	**Year**
QTL_1	1	1_4412249	4412249	G	A	0.204225	5.59727	1.13697	8.52334e-07	7.81	2018
QTL_2	2	2_1671507	1671507	C	T	0.101754	−6.31527	1.20641	1.65188e-07	8.74	2019
QTL_4a	4	4_1170622	1170622	C	T	0.140351	−5.79981	1.06439	5.06718e-08	9.41	2019
QTL_4b	4	4_13878965	13878965	C	T	0.157895	5.53481	1.1591	1.79631e-06	7.38	2018
QTL_13a	13	13_339517	339517	G	A	0.0982456	−7.08208	1.17641	1.74352e-09	11.25	2019
QTL_13b	13	13_4776228	4776228	A	G	0.249123	−4.25277	0.725704	4.62326e-09	10.72	2019
	13	13_4830850	4830850	A	G	0.412879	5.93241	0.945253	3.47396e-10	12.10	2018
QTL_14a	14	14_3385227	3385227	G	A	0.111888	−4.12006	0.807297	3.33384e-07	8.35	2019
QTL_14b	14	14_15012596	15012596	A	T	0.0496454	−7.26546	1.45255	5.67781e-07	8.04	2019
	14	14_15079728	15079728	C	G	0.101754	−9.63339	1.96973	1.00475e-06	7.72	2018

*^a^Reference allele present in CDC Bethune.*

*^b^Alternative allele.*

*^c^Minor allele frequency.*

*^d^Random polygenetic effect following a normal distribution.*

*^e^Proportion phenotype variance explained by a given SNP computed as proposed by (Shim et al., 2015)*.

### Candidate Gene Identification

Significant GWAS associations were further investigated for the presence of potential candidate genes using local linkage disequilibrium. For each QTL, the target region was defined as the region in linkage disequilibrium (r^2^ ≥ 0.2) with the most significant SNP (Supplementary Figure S9). A total of 102 candidate genes, based on the annotation of the reference genome CDC Bethune, were identified in these target regions corresponding to the 8 QTLs (Supplementary Table S8). Among them, genes known to be involved in plant immunity were identified for QTL_4b (LRR protein), QTL_13a (LysM-RLK protein), QTL_13b (RPW8), QTL_14a (RLK protein), and QTL_14b (RLK and PR proteins).

## Discussion

### SNP Discovery Pipeline

A panel of 311 genotypes was sequenced, and the sequence reads were mapped on CDC Bethune reference genome V2.0. The latest version of this reference genome integrates an optical map (Aston et al., 1999) as a guide for the assembly of the flax genome. This notably contributed to correct the positioning of certain scaffolds, the assembly of the scaffolds, and even the lack of positioning of certain scaffolds of the first version proposed by (Wang et al., 2012). Thus, this reference genome sequence totals 318 Mb in 88,384 unordered scaffolds and represents about 81% of the total flax genome.

A total of 5,464,275 SNPs were obtained on the entire flax panel including the two wild accessions, 1,693,910 SNPs being informative when considering the cultivated flax germplasm (after discarding low-quality and low-frequency SNPs). SNP density varies depending on chromosomes and their positions along the chromosome with a higher density observed on chromosomal arms than in central position. A similar SNP distribution along the 15 chromosomes was observed in other studies based on different flax diversity panels (Guo et al., 2020; Hoque et al., 2020). Despite high stringency criteria used, the SNP discovery pipeline exhibited a very high recovery rate of previously developed KASP SNP markers. While being of high quality, the set of SNPs discovered covers extensively the genome. About 14.7% of unknown nucleotides still remain on the CDC Bethune V2.0 FASTA sequence, as shown in Figure 1 (e.g., on chromosome 11, where the white area corresponds to unknown nucleotides between positions 703,455 and 1,218,107 bp of the chromosome). Therefore, no SNP detection could be performed in these non-covered regions. These sequence absences could be due to a high density of repeated sequences such as transposable elements which are mainly located at the ends of chromosomal arms in flax (You et al., 2018), or inter- and intra-chromosomal genome duplication events. Previous results suggest that flax has undergone one paleo-polyploidization event (23.8–44.1 million years ago) and one meso-polyploidization event (3.7–6.8 million years ago), followed by rearrangements, and deletions or fusion of chromosome arms from an ancient progenitor with eight haploid chromosomes (Jiao et al., 2011; You et al., 2018; Zhang et al., 2020). In CDC Bethune V2.0 assembly, a near complete synteny between chromosomes 2 and 13 was observed, whereas the other chromosomes displayed micro-syntenies between chromosomes 6 and 12 or between chromosomes 7 and 9 (You et al., 2018).

The highest number of private SNPs identified in oil flax (19.4% of total SNPs detected) suggests that this group could be the ancestor of cultivated flax, confirming previous analyses in flax (Soto-Cerda et al., 2013; Guo et al., 2020; Hoque et al., 2020). The two wild flax included in the panel provided 13.6% of the total SNPs detected. If we know the identity of one of these 2 accessions (*L. bienne*), the other is currently undetermined.

### Population Structure and Linkage Disequilibrium

The slight prevalence of fiber flax in our diversity panel (188 accessions out of 311) showed a new population structure for this flax type in comparison with previously published results. Indeed, analysis of the genetic structure of this panel reveals four distinct genetic clusters: two fiber clusters (with mostly flax originated from Eastern Europe and from Western Europe) and two oil clusters (with mostly flax from America-Europe and from Asia/Middle-East). At k = 4, the clustering highlights a geographical distribution linked to flax type. Note that for k = 6, the two additional groups highlight the recent breeding history (minimum BIC value for k = 6, Supplementary Figure S4). Indeed, the AM-EU and E-EU clusters are each divided into two subgroups discriminating specifically Canadian vs. AM-EU oil flax and Russian vs.E-EU fiber flax, respectively. In addition, the analysis performed with the complete diversity panel assigned the two wild flax into two additional distinct groups, each consisting of a single individual per group.

Here, we observed a rapid LD decay in cultivated flax (20.43 kb for a r^2^ threshold of 0.2) and a steeper decay in oil group than in fiber groups (11.82 and 39.57 kb, respectively, for a r^2^ threshold of 0.2). In a recent Chinese study on a diversity panel, of similar composition (size, balanced in fiber / oil), a 50% decrease of DL was reached at a physical distance equals to 6.4 kb for the oil group and 25.3 kb for the fiber group (Guo et al., 2020). By comparison, in our study, 50% drop in LD is observed at about 3.15 and 14.07 kb in the oil and fiber flax group, respectively. This faster decrease of DL in our diversity panel could be the result of a greater genetic diversity in the two main types of flax. This facilitates the search for candidate genes through efficient narrowing of putative QTL regions. Due to its small size (22 individuals) and the large LD extent in this group, we decided to remove the oil AS-ME cluster from the GWAS analyses.

### GWAS Identifies RPW8 Genes and RLK in Powdery Mildew Resistance

A total of eight QTLs located on chromosomes 1, 2, 4, 13, and 14 were significantly associated with resistance to *Oïdium lini*. QTL_13b had previously been identified in QTL linkage analyses based on two connected recombinant inbred line populations (A. Speck, pers. Com.), thus confirming the robustness of this QTL and the reliability of the current GWAS analyses. In this study, QTL_13b and QTL_14b had robust effects over the 2 years, whereas the other 6 QTLs were detected only in one of the 2 years.

None of the 8 QTLs identified in this study have been previously reported in Asgarinia et al. (2013) and consequently correspond to new loci. Asgarinia et al. (2013) identified 3 powdery mildew resistance QTLs located on chromosomes 1, 7, and 9 using an F2 population derived from a cross between the susceptible oil cultivar Norman and the resistant oil cultivar Linda. However, we could not reproduce these results in French fields using a similar F2 Norman x Linda population (A. Speck, com. pers.). This strongly suggests that different races of *Oïdium lini* are present in Canada and France. Consequently, we assume that the 3 QTLs reported in Asgarinia et al. (2013) were not identified in the present GWAS analysis, not only because the corresponding genes are not present in our panel, but also because the races of *O. lini* are different.

The analysis of DL between the 10 most significant SNPs revealed a strong correlation between QTL_2 and QTL_13a (r^2^ = 0.96). It has to be noted that QTL_2 was supported by only one SNP with a significant effect. The strong LD between this SNP and the SNPs supporting QTL_13a makes us believe that QTL_2 might not correspond to an actual QTL but either (i) to a wrong attribution of this SNP marker to chromosome 2 or (ii) to a long-range LD not sufficiently controlled in the GWAS model by the kinship and the population structure. Further investigations are therefore needed to verify this QTL.

Disease severity was scored during the flowering period in 7-day intervals, highlighting a temporal dynamic of immune responses in flax. QTL_13b explained a higher proportion of the phenotypic variance at the early stage of powdery mildew attack and its effect decreased during the weeks of scoring. This decrease of QTL_13b effect was compensated by the increase of other QTLs effects (QTL_1, QTL_4a, QTL_14a, and QTL_14b).

Until recently, breeding for disease resistance has been mainly based on major resistance (R) genes, most of them encoding nucleotide-binding and leucine-rich repeat proteins (NLR). NLR-mediated resistance is strong but is unfortunately often rapidly overcome by virulent strains of the pathogens. In that context, quantitative resistance is gaining increasing attention for breeding purpose. In this study, 8 QTLs were identified and none of the target region harbors NLR genes. Accordingly, resistance QTLs are known to encode a great diversity of function (Pilet-Nayel et al., 2017). Even if the target regions remain quite large in this study (from 20 to 100 kb), it was able to pinpoint few interesting candidates. A number of two tandem RPW8 genes were present in the target region of QTL_13b (Supplementary Table S8). In Arabidopsis and rice, RPW8.1 and RPW8.2 genes are known to confer broad-spectrum resistance to powdery mildew (Xiao et al., 2001; Wang et al., 2009; Berkey et al., 2017; Li et al., 2018), suggesting that *linum* RPW8 genes are the strong candidates for QTL_13b. We also identified other interesting candidate genes encoding LysM-RLK (QTL_13a), RLK (QTL_14a and QTL_14b), and PR (QTL_14b), known to be involved in the perception and defense against microorganisms (Buendia et al., 2018; Dievart et al., 2020; Gottin et al., 2021).

### Use of Various Sources of Resistance in Breeding Programs

Historically, flax farming in Western Europe took place from spring to summer, but recently, winter flax farming has considerably grown. The consequence of this is to make an almost complete continuum of flax cultivation during the 12 months of the year, thus generating an earlier primary inoculum of the fungi. One strategy to counteract this primary inoculum would be to use different powdery mildew resistance genes between spring and winter flax varieties. The implementation of this strategy implies to know precisely the origins of the resistance. In our analysis, minor allele frequency (MAF) distribution of the most significant SNPs varies according to the four genetic groups that we identified. If the alleles associated with resistance at snp1_4412249 (QTL_1), snp4_13878965 (QTL_4b), snp13_4776228 (QTL_13b), and snp13_4830850 (QTL_13b) are present in the four genetic groups, the other ones are absent either from the Western Europe fiber group or from the Asia/Middle East oil group, or both. The present work therefore provides the information needed to implement this strategy in breeding (Table 2).

**Table 2:** Minor allele frequency (MAF) distribution among genetic groups of the most significant SNPs associated with powdery mildew resistance.

**QTL name**	**SNP**	**Oil AM-EU (%)**	**Fiber E-EU (%)**	**Oil AS-ME (%)**	**Fiber W-EU (%)**
QTL_1	1_4412249	24.49	23.70	59.09	3.92
QTL_2	2_1671507	22.68	5.11	0.00	0.00
QTL_4a	4_1170622	34.02	5.11	68.18	0.00
QTL_4b	4_13878965	3.06	28.47	22.73	6.00
QTL_13a	13_339517	21.43	5.11	0.00	0.00
QTL_13b	13_4776228	48.98	8.03	77.27	24.00
	13_4830850	15.56	47.06	9.09	81.58
QTL_14a	14_3385227	19.39	8.03	0.00	3.92
QTL_14b	14_15012596	7.45	5.11	13.64	0.00
	14_15079728	22.45	5.11	80.95	0.00

The second strategy would be to pyramid in varieties powdery mildew resistance genes with the help of marker-assisted selection. In the case of an introgression of powdery mildew resistance genes from oil flax into fiber flax, back-crossing steps would be necessary to keep the favorable genetic background of the flax fiber. This strategy can be further extended by genomic selection, allowing to raise the background resistance of flax germplasm due to QTLs of minor effect (Poland and Rutkoski, 2016), not detected by GWAS. Resistance genes pyramiding would allow the creation of new flax varieties with durable resistance (Mundt, 2018) corresponding to the current farmers' needs.

## Data Availability Statement

The datasets presented in this article are not readily available due to commercial restrictions. Requests to access the datasets should be directed to the corresponding author.

## Author Contributions

AS and J-PT performed the establishment of collections of flax germplasm and the phenotyping. AS analyzed data and wrote the manuscript with significant input from all authors. All authors read and approved the final manuscript.

## Funding

The authors declare that the study received funding from Terre De Lin Développement. The funder had the following involvement in the study: DNA extraction, libraries construction, sequencing, and field phenotyping.

## Conflict of Interest

AS and J-PT were employed by Terre De Lin. The remaining authors declare that the research was conducted in the absence of any commercial or financial relationships that could be construed as a potential conflict of interest.

## Publisher's Note

All claims expressed in this article are solely those of the authors and do not necessarily represent those of their affiliated organizations, or those of the publisher, the editors and the reviewers. Any product that may be evaluated in this article, or claim that may be made by its manufacturer, is not guaranteed or endorsed by the publisher.
