# Supplementary material for: Genetic Architecture of Powdery Mildew Resistance Revealed by a Genome-Wide Association Study of a Worldwide Collection of Flax (Linum usitatissimum L.)
Source: Front Plant Sci. 2022 Jun 24;13:871633. doi: 10.3389/fpls.2022.871633 (PMC9263915; doi:10.3389/fpls.2022.871633)
Supplement: Supplementary file 1 [file Data_Sheet_1.docx]

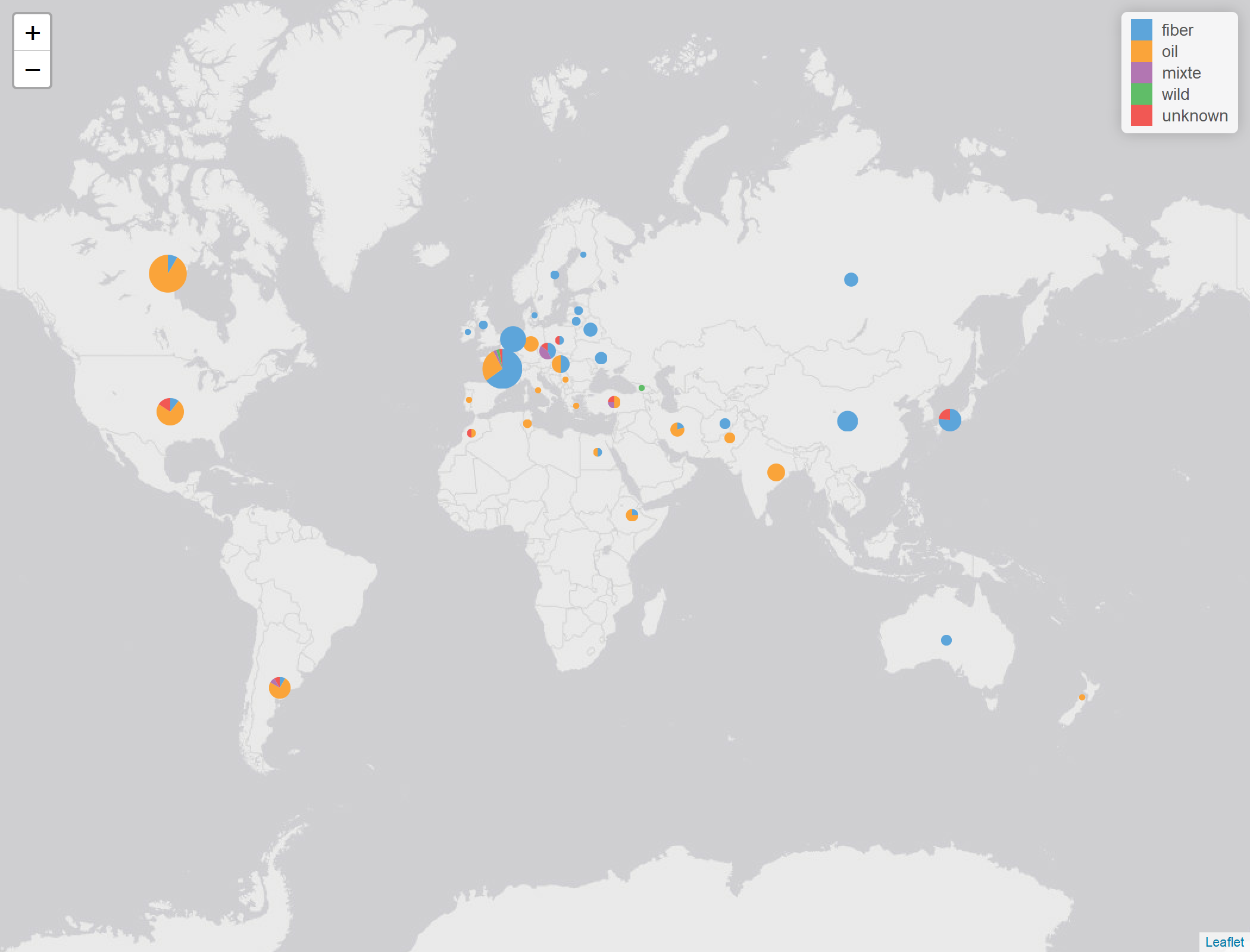


**Supplementary Figure S1**: Flax world distribution present in the diversity panel.


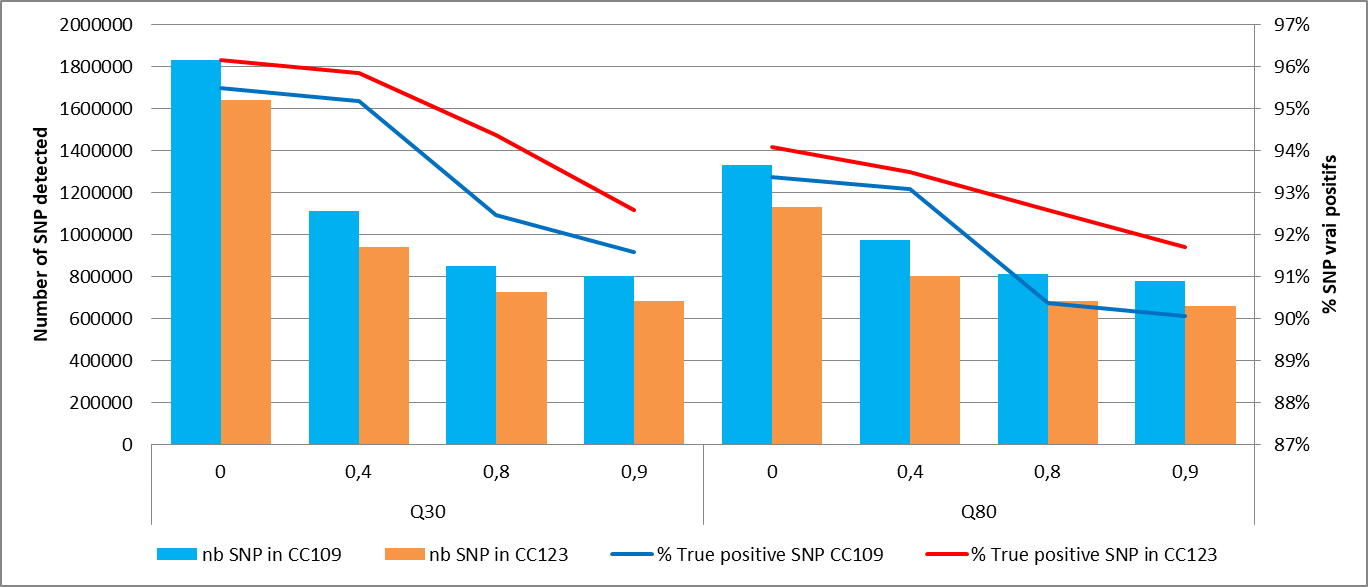


**Supplementary Figure S2**: True SNPs detected by the pipeline on 2 different genotypes (CC109 and CC123) according alignments quality (Q>30 and Q>80) and allele frequency (ranging from 0 to 0.9).

**
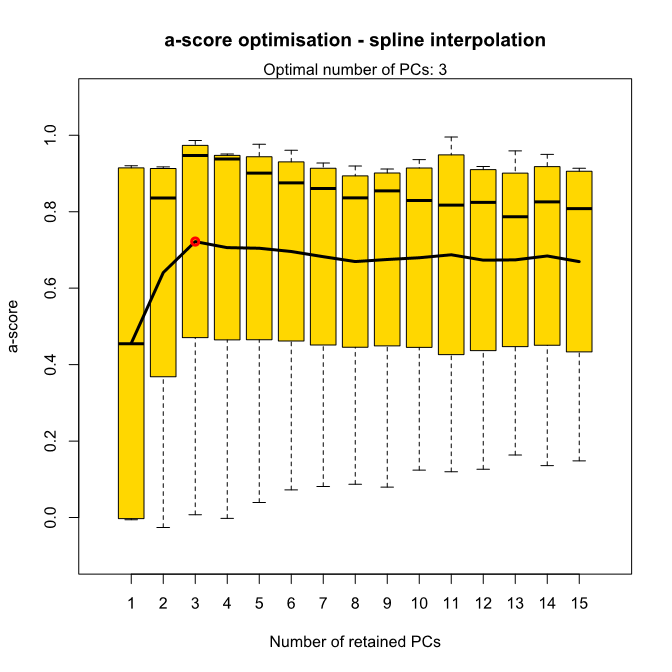
**

**Supplementary Figure S3:** a-score plot to determine the optimal number of ACP axis for the DAPC.


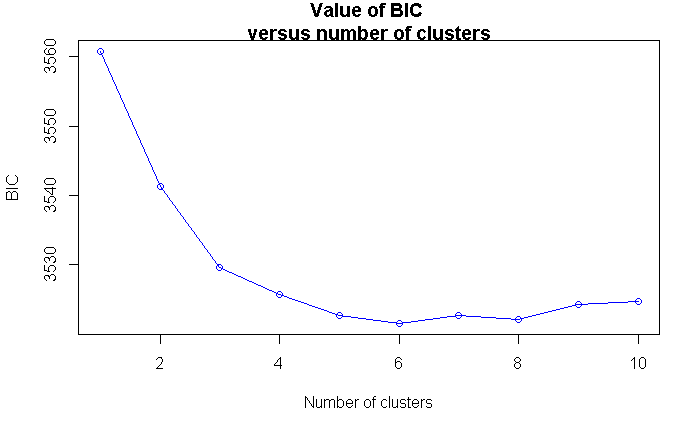


**Supplementary Figure S4**: Bayesian Information Criterion (BIC) value for increasing values of k (from 1 to 10).


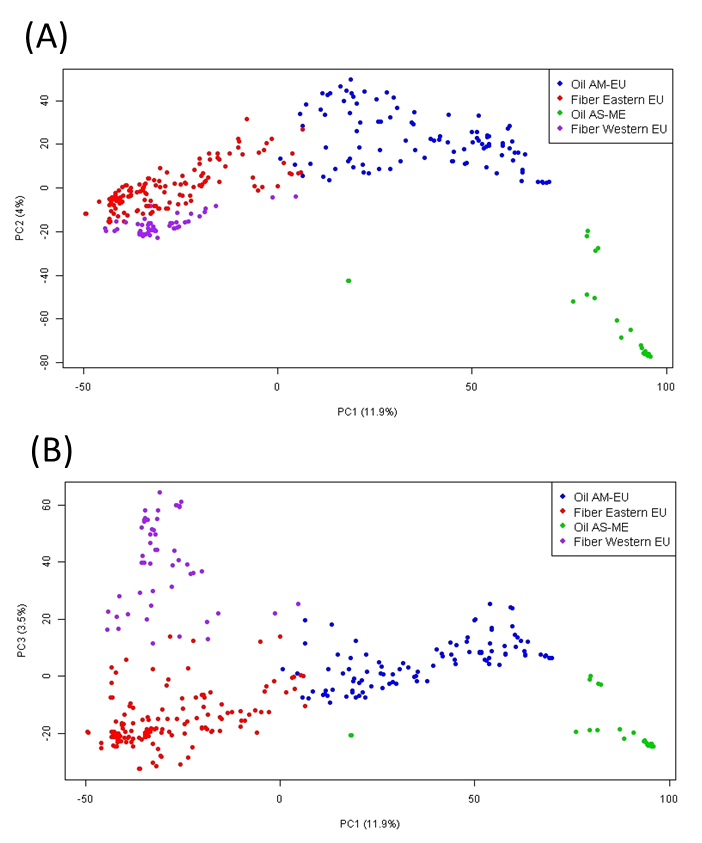


**Supplementary Figure S5**: Graphical plot of the principal component analysis (PCA) of the flax panel colored by the clusters identified by DAPC (k=4). (A): PCA axis 1 and 2, (B): PCA axis 1 and 3.


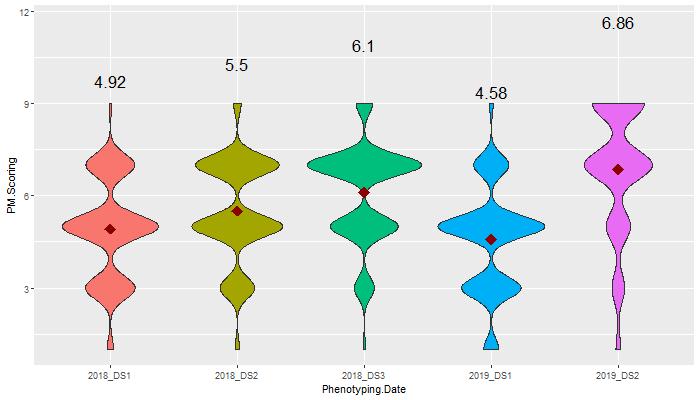


**Supplementary Figure S6:** Temporal evolution of powdery mildew in 2018 and 2019 on the flax diversity panel. Scale from 1 (0-5% infected leaf area) to 9 (75-100% of infected leaf area).


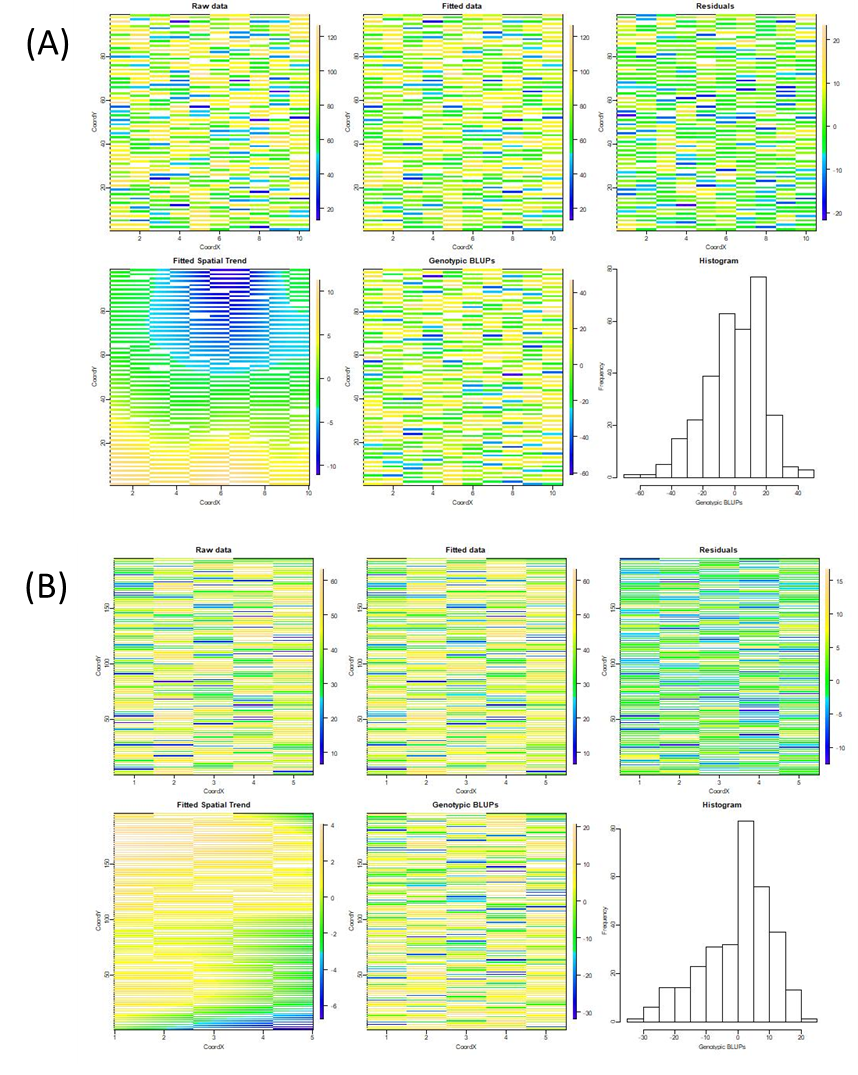


**Supplementary Figure S7**: The following graphics are depicted from top left to bottom right (A: 2018 year and B: 2019 year): the raw data, the fitted data, the residuals, the estimated spatial trend, the genotypic BLUEs and their histogram. Except for the histogram, the plots are depicted in terms of the spatial coordinates (e.g., the rows and columns of the field).


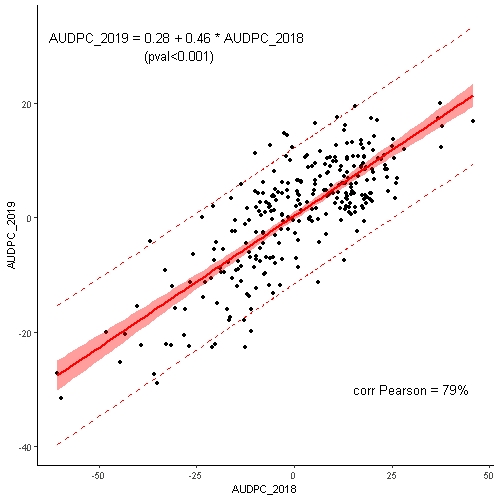


**Supplementary Figure S8**: Correlation between AUDPC 2018 and 2019 by accession after correction of spatial effects by SpATS R-package.


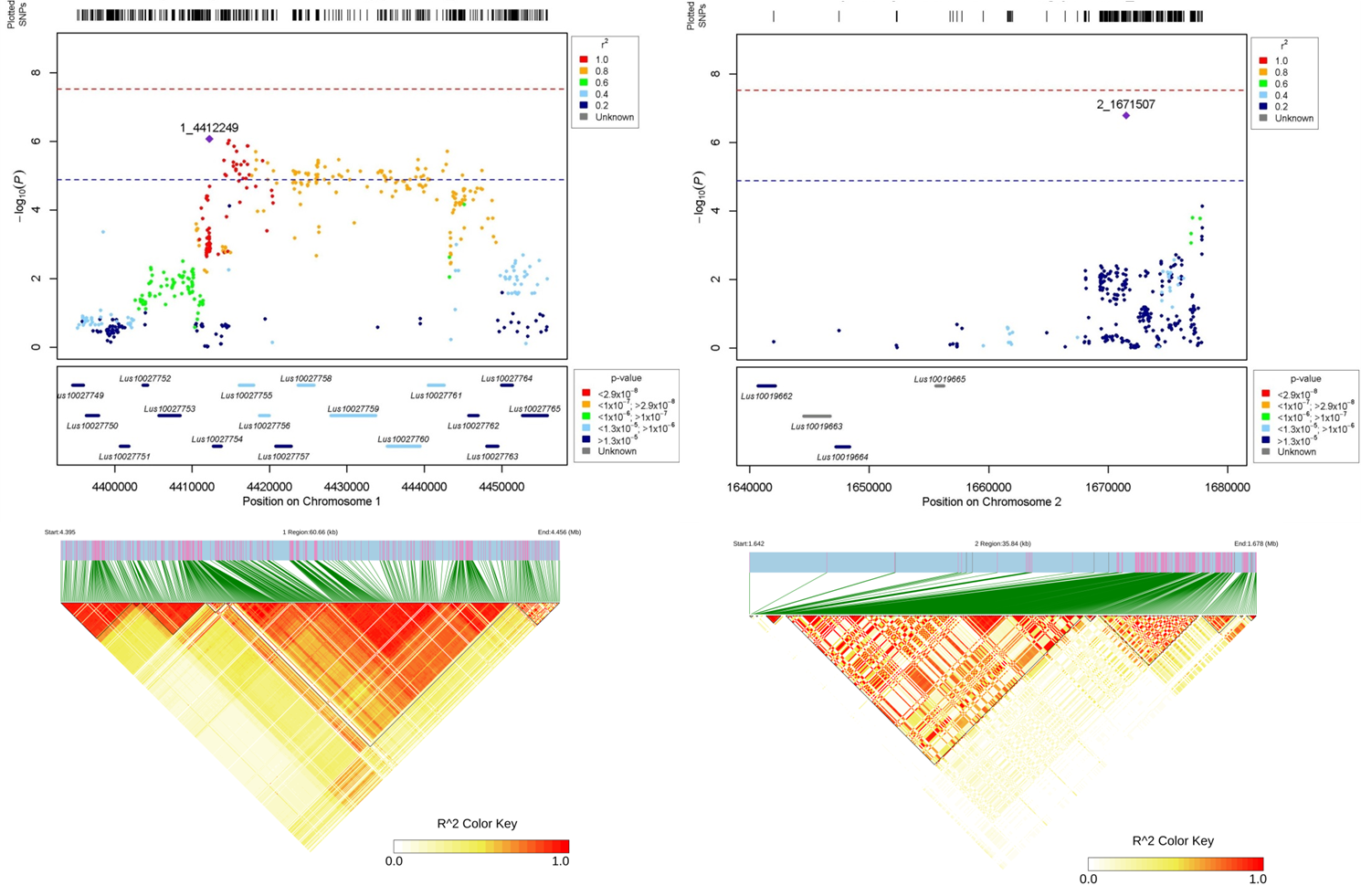


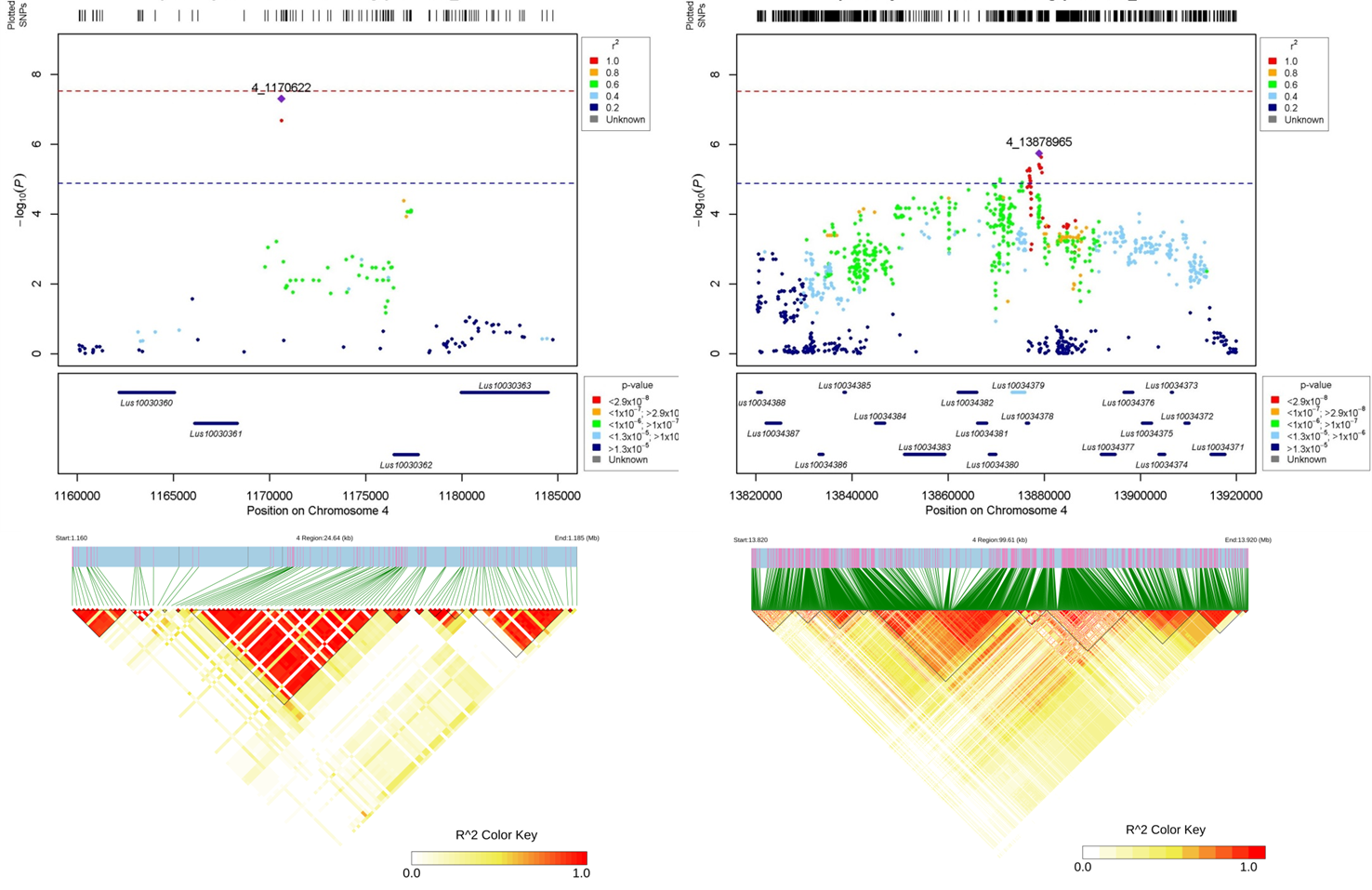


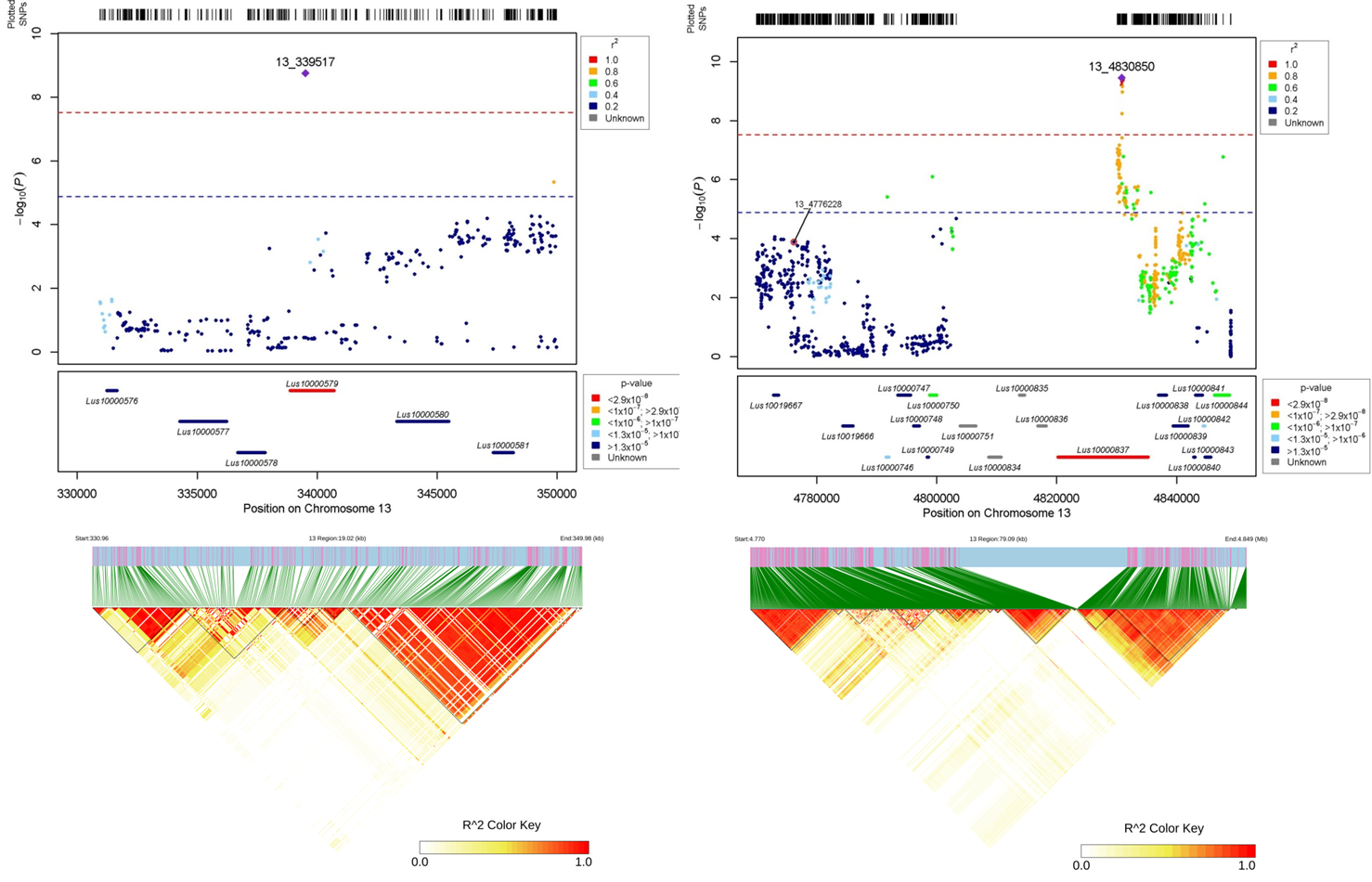


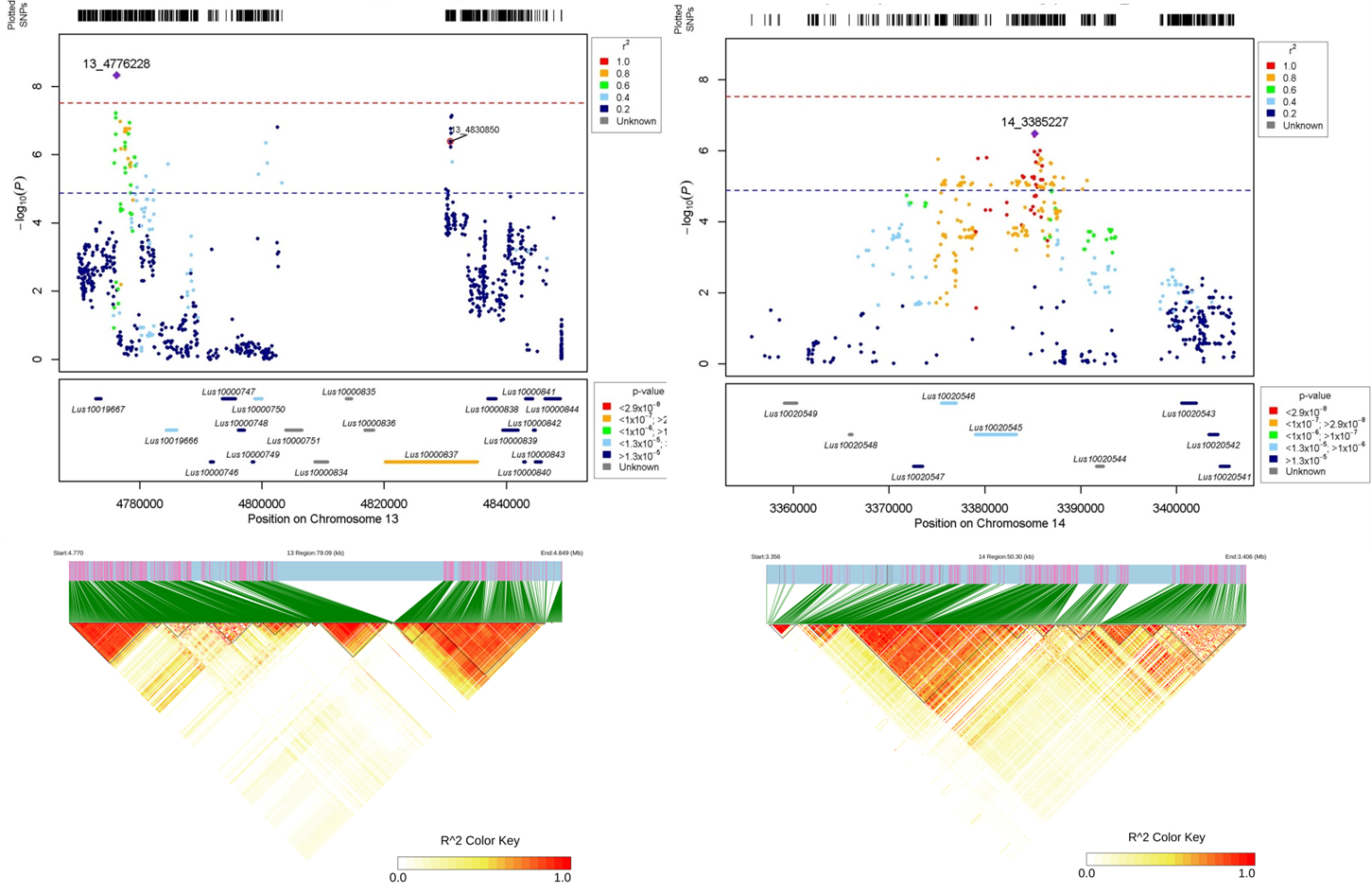


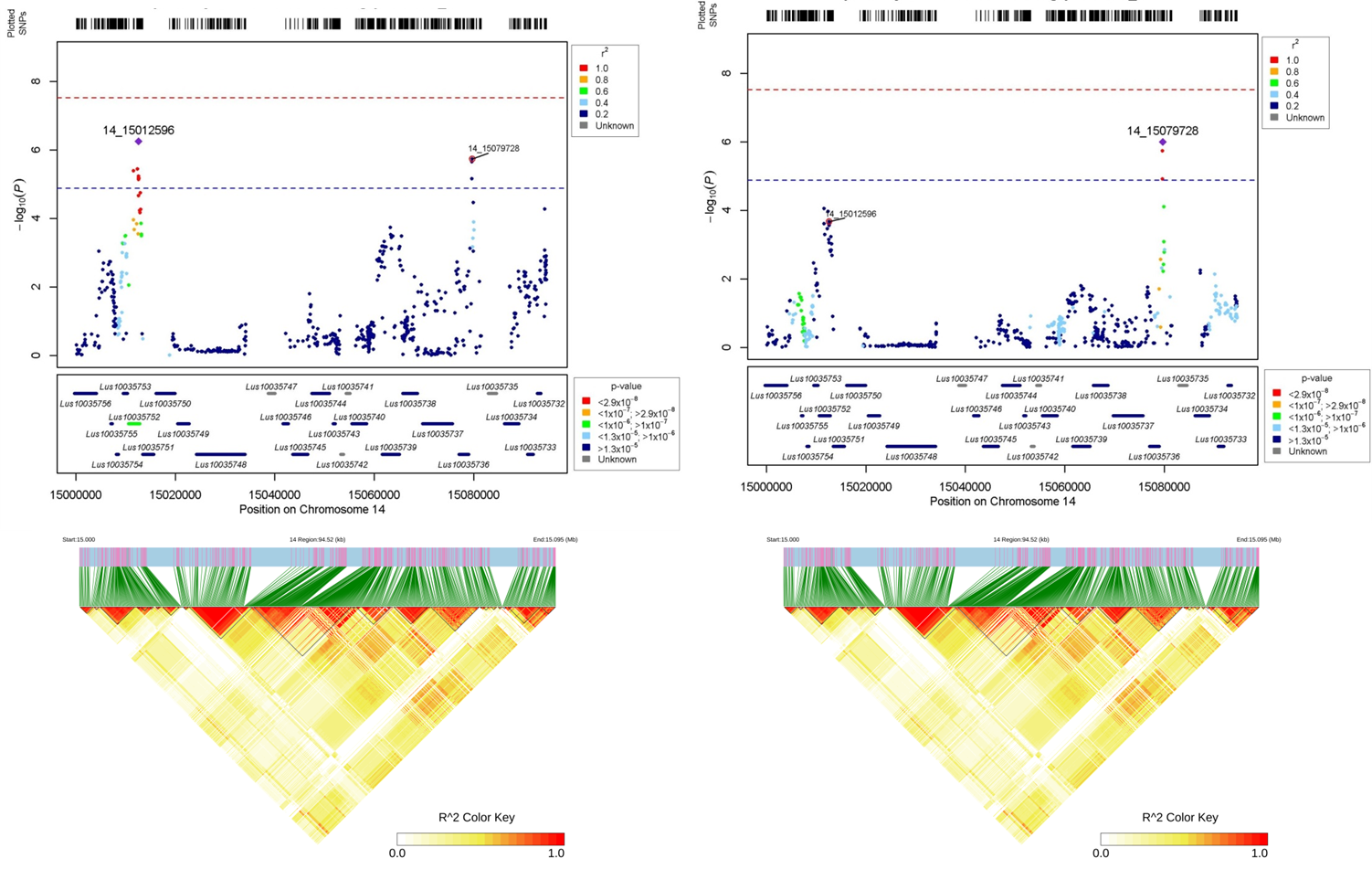


**Supplementary Figure S9:** Local association plot of the 8 QTLs identified in flax. SNPs and genes are color coded based on linkage disequilibrium with the top marker and GWAS p-value respectively. The red line shows the Bonferroni-corrected genome wide threshold and the blue line shows the FDR threshold.
